# Development of the Rat Model of Lapatinib-Induced Diarrhoea

**DOI:** 10.1155/2014/194185

**Published:** 2014-07-07

**Authors:** Joanne M. Bowen

**Affiliations:** School of Medical Sciences, University of Adelaide, Frome Road, Adelaide, SA 5005, Australia

## Abstract

Targeted therapy of cancer is often associated with clinically significant diarrhoea; however, the mechanisms underpinning this adverse effect are currently unknown. Diarrhoea following treatment with tyrosine kinase inhibitors (TKIs) of EGFR is particularly troublesome. Until recently, understanding of EGFR TKI-induced diarrhoea has been limited to clinical observation. However, our group has recently developed the first rat model of EGFR TKI-induced diarrhoea. This paper reviews the published and unpublished findings.

## 1. Introduction

Molecularly targeted cancer agents are emerging as an effective approach to the treatment of a range of different tumours. They have ushered in a new age of personalised medicine in cancer therapy and have been made possible due to significant advances in genetics and molecular biology in the last two decades. However, despite improved specificity for cancerous cells compared to traditional cytotoxic chemotherapy, these agents are still associated with a number of important clinical adverse effects, particularly rash and diarrhoea. Importantly, the mechanisms underlying these toxicities and the significance to patient outcomes are poorly understood and have received little attention to date. In response to this gap in knowledge, our research group has developed a clinically relevant rat model of diarrhoea in response to treatment with the small molecule tyrosine kinase inhibitor (TKI) of epidermal growth factor receptor (EGFR) and human epidermal growth factor receptor (HER2), lapatinib. This review will reflect on the rationale for embarking on the project, the series of experiments that established the model, and the challenges encountered along the way.

## 2. The Importance of Diarrhoea as an Adverse Effect of Targeted Cancer Therapy

Diarrhoea is a troublesome side effect of almost all systemic cancer treatments and has been researched intensely [1–3]. Whilst there is improved understanding of the pathobiology of diarrhoea following traditional chemotherapy agents, the same is not true for targeted cancer agents. The reasons are multifactorial and include targeted therapies being a relatively recent addition to the arsenal of cancer therapies, the speed at which these new drugs emerge, and the diversity of targets for each agent. There are a number of different classes of targeted therapy agents, with arguably the most established being the small molecule inhibitors of receptor tyrosine kinases (TKIs). General subclasses of TKIs have been based on the specific molecules targeted, including EGFR, VEGFR, and PDGFR to name a few, although in reality most agents are multitargeted and selectively promiscuous for off-target kinases [4]. This creates significant complexity when elucidating mechanisms of adverse effects due to TKIs. What is more, TKIs may be administered alone or in combination with conventional chemotherapy agents or radiotherapy. These multimodal regimens may compound or act synergistically on normal tissue damage, making it difficult to separate the role of TKIs on injury and, consequently, manage resulting symptoms.

Diarrhoea is one of the most common adverse events recorded following treatment with all TKIs and is a dose limiting toxicity for TKIs that block EGFR signalling [5]. Diarrhoea is an important cause of therapy interruption and negatively affects compliance [6]. Diarrhoea theoretically may also impede full dosage of orally administered agents [7], although any influence on pharmacokinetics and pharmacodynamics has yet to be investigated. In patients treated with TKIs targeting EGFR, diarrhoea is an important clinical toxicity which is worsened when combined with chemotherapy. In phase III trials of EGFR TKIs, gefitinib, and erlotinib, diarrhoea was found second only to rash as the most commonly reported adverse event [8]. In a recent meta-analysis of multiple EGFR TKIs and nonsmall cell lung cancer (NSCLC), it was found that severe grade diarrhoea was increased significantly by addition of chemotherapy [9]. In a meta-analysis of phase II studies of EGFR TKIs in head and neck squamous cell carcinoma (HNSCC), diarrhoea occurred in 45% of patients. Interestingly, diarrhoea was also associated with clinical benefit defined as complete response, partial response, or stable disease (OR = 1.77, 95% CI = 1.05–2.97) and overall survival (HR = 0.65, 95% CI = 0.51–0.83) [10]. Associations between diarrhoea and treatment response require further investigation, although it is plausible given the established link between rash and response to EGFR inhibition [11].

A summary of diarrhoea associated with TKIs for EGFR is shown in Table 1.

## 3. What Is Known about TKI-Induced Diarrhoea?

Despite diarrhoea being extremely common and in some cases dose-limiting, there has been almost no clinical research investigating the underlying causes of this side effect. Possible reasons may include the misconception that diarrhoea is an unimportant toxicity combined with the difficulties in directly assessing the gastrointestinal tract. Although there is yet to be any defined pathogenesis, some authors cautiously speculate that TKI-induced diarrhoea is a specific toxicity separate from chemotherapy-induced mucosal injury [15], which arises from lesions within the small intestine [16]. In the instance of TKI monotherapy, studies have shown that diarrhoea is dose dependent rather than associated with pharmacokinetics [17, 18], suggesting that the toxicity is predominantly caused by luminal exposure to the drug.

EGFR is expressed by cells of epithelial origin, including the skin and gastrointestinal tract, so it is not surprising that toxicities affect these systems. The prevailing hypothesis is that inhibition of EGFR signalling will lead to reduced growth and healing of the intestinal epithelium, leading to mucosal atrophy, due to the stimulatory effect of the EGFR pathway on enterocyte proliferation [19]. An alternative theory implicates excess chloride secretion caused by altered EGFR signalling to downstream pathways and channels [20]. This is emerging as a favoured explanation due to the known inhibitory effects of EGF on chloride secretion in the intestine and the understanding of the profound importance of chloride movement in secretory diarrhoea [21].

Diarrhoea is often worsened during TKI and chemotherapy combination treatment and may be due to shared metabolic and drug efflux pathways between drugs. For example, coadministration of lapatinib and paclitaxel results in 20% increased exposure to both agents, suggested to be through downregulation of CYP3A4 [22]. Pazopanib downregulates UGT1A1, so use with drugs that are substrates for the enzyme, such as irinotecan, may increase toxicity [23]. Both TKIs and chemotherapy agents are substrates for the drug efflux transporters of the ATP Binding Cassette family and each has the potential to inhibit clearance of the other drug, leading to drug accumulation, an important cause of increased toxicity [24]. When TKIs are combined with radiotherapy, there may be reduced tissue healing by inhibition of growth factors in response to radiation, in addition to enhanced apoptosis. These are all plausible explanations but have yet to be proven given that there is no direct research completed to date.

Recognising and understanding this major lack of research, we decided to establish a platform to investigate mechanisms and test interventions for EGFR TKI-induced diarrhoea which led to the development the first rat model of targeted therapy-induced diarrhoea.

## 4. Development of a Model to Study TKI-Induced Diarrhoea

The use of animal models to investigate treatment-related toxicity has provided a wealth of knowledge in regard to injury pathogenesis and has enabled relatively high throughput testing of interventions matched to pathobiology, which has been particularly evident in the mucositis field [25–27].

In contrast, there is a paucity of models available to specifically investigate mechanisms of TKI-induced diarrhoea. As such, our group has developed a rat model of targeted therapy-induced diarrhoea that uses a four-week schedule of daily oral lapatinib treatment to induce mild to moderate diarrhoea, whilst achieving a C_min⁡_ similar to clinical studies. Conventionally housed male albino Wistar rats received an oral gavage of study drug (TKI) or vehicle (0.5% methylcellulose/0.1% Tween 80) every 24 h for 28 days. Daily measurements of weight loss, diarrhoea, and distress were recorded. Groups of rats were sacrificed each week to assess circulating lapatinib levels and gastrointestinal tissue changes at the morphological and molecular level. Full experimental details can be found in Bowen et al. [28].

Lapatinib (GW572016/Tykerb GlaxoSmithKline) was chosen as the initial TKI for testing in the model since it is a highly promising drug in clinical trials for the treatment of multiple solid tumours [22], but optimal use is limited by diarrhoea. Lapatinib is an orally administered small molecule TKI targeting ErbB-1 (EGFR) and ErbB-2 (HER2) [29]. Lapatinib's anticancer effect is mediated through inhibition of HER2 in overexpressing tumours, preventing downstream signalling to extracellular signal-related kinase- (ERK-) 1/2 and phosphatidylinositol-3′-kinase (PI3K)/Akt pathways. ERK and PI3K have numerous roles within the cell primarily concerning growth, proliferation, and survival [30]. Lapatinib was first approved for treatment of HER2 positive advanced and metastatic breast cancer in combination with capecitabine in patients who have previously received anthracycline, taxane, and trastuzumab [31]. Lapatinib is now also approved for treatment of postmenopausal women with hormone receptor positive metastatic breast cancer that overexpress HER2 and for whom hormonal therapy is indicated. Diarrhoea is the most common adverse event associated with lapatinib [32]. A pooled analysis of phases II and III studies of lapatinib showed that 51% of patients experience diarrhoea [12]. Addition of lapatinib to capecitabine increased diarrhoea from 30% to 65%, whilst addition of lapatinib to paclitaxel increased diarrhoea from 28% to 48%. Not only was diarrhoea incidence and severity increased, but also it occurred with earlier onset to chemotherapy alone and was the most common cause of dose reduction. In the recent NeoALLTO trial, 23% of patients receiving lapatinib combined with paclitaxel before definitive surgery experienced severe grade diarrhoea [33]. No significant association was observed between diarrhoea and pathologic complete response [34]. As such, diarrhoea is an important cause of poor treatment outcomes.

**Figure 1:**
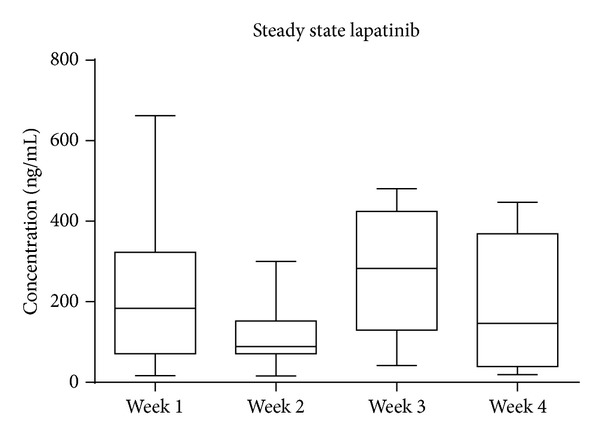
Circulating lapatinib measured weekly. Data shown is median with range, *n* = 12. Steady state lapatinib levels were determined by LC/MS/MS and remained similar across the four weeks of treatment (*P* > 0.05, one-way ANOVA).

In our rat model, we found that lapatinib in a dose range of 100 to 500 mg/kg induced diarrhoea in a majority of rats, without being excessively toxic over the month of treatment. At 240 mg/kg lapatinib, roughly 70% of rats experienced diarrhoea during the treatment period, with diarrhoea episodes characterised by intermittent and repeated presentation [28]. This reflects what is seen clinically and uniquely positions the model for investigation of interventions. Furthermore, circulating lapatinib concentrations remained at a clinically relevant [16, 35] steady state throughout the treatment period (Figure 1). Pharmacokinetic studies have consistently found that lapatinib exposure varies considerably between patients [36], which was also noted in our rat study. The causes for this variability are currently unknown but may be influenced by the presence of food in the stomach which has been shown to increase bioavailability of lapatinib [36]. In our study, rats were not fasted before or after administration of lapatinib which may have contributed to the observed variation in steady state levels.

Although our model created symptomology similar to the clinical situation, somewhat surprisingly there was no macroscopic or microscopic tissue injury seen within the jejunum or colon. As such, lapatinib does not cause epithelial atrophy at clinically relevant doses in the rat, and the mechanism of diarrhoea does not rely on this change occurring. In contrast to these findings, studies in mice examining various EGFR TKIs have shown gastrointestinal damage with significant epithelial atrophy. Mice administered gefitinib twice daily for ten days had prominent changes in the small intestine [37]. The authors concluded that the appearance of the small intestine was consistent with necrotic enterocolitis due to inhibited intestinal homeostasis and healing. A study that treated mice for nine days with the pan-ErbB inhibitor, canertinib, showed that treatment resulted in decreased villus height and small intestinal wet weight [38]. Lastly, erlotinib, administered as an intraperitoneal injection daily for ten days, caused reduced small intestinal weight and villus height, and this was worsened when given in combination with cisplatin [39]. In each model, cotreatment with GLP-2 or other growth factors including KGF or EGF could reverse the atrophy caused by EGFR TKIs. It is unclear whether the dose and schedule selected in these studies recapitulated the clinical effects of diarrhoea and if circulating levels of the drugs were within a human equivalent range. However, it does uncover the potential issue of species differences in response to TKIs between mice and rats.

## 5. Addition of Chemotherapy to the Model of TKI-Induced Diarrhoea

**Figure 2:**
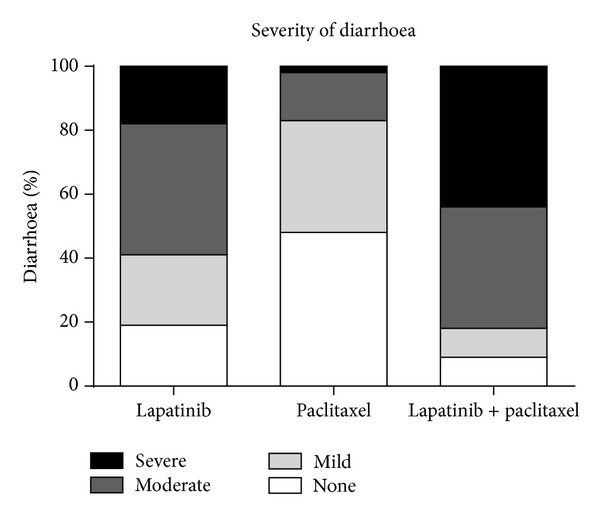
Diarrhoea incidence and severity. Data shown is proportion of rats that experienced diarrhoea at each severity level measured over the duration of treatment, *n* = 30. Addition of paclitaxel to lapatinib treatment caused a significant increase in the proportion of rats that experienced severe grade diarrhoea (*P* < 0.001, chi-square test).

Our rat model of TKI-induced diarrhoea also assessed the effect of combination therapy with paclitaxel due to clinically important diarrhoea seen in patients treated with both lapatinib and chemotherapy. Paclitaxel was chosen as the chemotherapy agent to investigate in our model since it is the current drug of choice for neoadjuvant breast cancer therapy [33]. Paclitaxel (Taxol) is a microtubule stabilisation agent of the taxane class and is considered moderately mycotoxic [40]. We found that combined treatment with paclitaxel and lapatinib caused a significant increase in the proportion of rats with severe diarrhoea (Figure 2) and also caused weight loss indicating clinically important intestinal injury in our model. Furthermore, combined lapatinib and paclitaxel treatment resulted in increased circulating lapatinib levels, which again reflects what is seen clinically [22]. Although the impact of paclitaxel treatment is likely the main contributor to increased gastrointestinal toxicity, we are unable to discount the role of absorbed lapatinib on diarrhoea entirely. Although most investigators suggest that unabsorbed drug is the cause of lapatinib-induced diarrhoea, clinical studies of erlotinib have shown an association between circulating drug levels and toxicity [41, 42]. Further investigation is needed to assess changes in drug exposure following concurrent treatment regimens and the effect on diarrhoea.

Given that we found no measurable intestinal pathology following lapatinib treatment alone, our study did not support the theory of TKI-induced direct mucosal damage leading to diarrhoea [15]. It is well established that treatment with conventional chemotherapy drugs causes atrophy of intestinal mucosa leading to mixed secretory/osmotic-type diarrhoea due to an inability to control solute absorption and secretion through decreased surface area [2]. It is clear that lapatinib-induced diarrhoea is occurring through a different mechanism. The alternative hypothesis is that lapatinib alters chloride secretion into the gastrointestinal lumen by interfering with inhibitory signals mediated through EGFR [15]. Our study does not support this either since blood biochemical analysis found that 240 mg/kg lapatinib had no significant effect on serum chloride. As such, the role of chloride secretion, or other ion transport mechanisms in lapatinib-induced diarrhoea, still remains unclear.

In contrast, combined lapatinib and paclitaxel treatment did cause gut tissue alterations, including epithelial apoptosis, crypt hyperplasia, and inflammatory infiltrate to the lamina propria. Previous research by our group using conventional chemotherapy drugs including methotrexate, 5-fluorouracil, and irinotecan has shown that apoptosis is a key early marker of chemotherapy-induced gastrointestinal injury, and crypt hyperplasia occurs during the regenerative phase following severe injury [26, 43–46]. Our rat models of diarrhoea due to acute chemotherapy treatment and the similarities in marker profiles in the current model provide evidence that paclitaxel combined with lapatinib caused intestinal injury, albeit relatively mild severity. Of particular interest is the chemosensitzing effect of lapatinib to paclitaxel on the small intestine. Our study found no effect of paclitaxel alone or lapatinib alone on small intestinal weight, crypt hyperplasia, or apoptosis, whereas combined treatment increased each of these outcomes significantly [28]. It should also be noted that changes in these parameters in the large intestine were not observed following lapatinib with or without paclitaxel. This supports the suggestion that lapatinib-induced diarrhoea seen in clinical trials is caused by damage localised to the small intestine [16] and indicates that this should be the focus of intervention studies. The more pronounced effects on the small intestine relative to the colon are likely a factor of greater exposure of the mucosal surface to unabsorbed drug in the proximal intestine. Furthermore, the relatively higher rate of epithelial turnover also increases susceptibility of the small intestine to the cytostatic and cytotoxic effects of conventional chemotherapy agents.

## 6. Future Directions

Development of this model is the first step in creation of a clinically relevant platform to examine mechanisms and test treatments for EGFR TKI-induced diarrhoea. We have built upon our group's collection of established chemotherapy-induced models of diarrhoea and believe this is a significant addition to the field. However, our preliminary findings have identified a number of aspects that need further clarification to improve our understanding and management of lapatinib-induced diarrhoea. Firstly, given that lapatinib bioavailability is relatively low and highly variable between patients [36], a close examination of factors that regulate gastrointestinal absorption is needed. This should include the pharmacokinetic effect of coadministration of antimotility agents used to manage diarrhoea. This research will improve dose tailoring for individuals, leading to improved drug safety and efficacy. Secondly, to further increase the utility of the model it should be isogenic and tumour bearing. This will allow evaluation of interventions for lapatinib-induced diarrhoea in rats that are not immunologically altered, whilst ensuring that there is no tumour protection. Finally, future research should concentrate on expanding the number of agents used in combination with lapatinib in this model. To date we have only assessed lapatinib together with a single chemotherapy drug. However, EGFR inhibition alongside radiation therapy is a proven clinical approach to head and neck cancer [47]. As such, this model could be adapted for localised radiotherapy, multiagent chemotherapy, or any combination thereof, with lapatinib.

The outlook for the clinical use of TKIs is to increasingly include them in first line treatment of curable disease. Therefore, it is extremely important to have a thorough understanding of the adverse effects associated with their use and the best approaches to minimise toxicity. Animal models such as the one described herein are an invaluable source of knowledge and will provide the platform to conduct studies that improve our knowledge of the mechanisms underlying diarrhoea as well as to uncover the best intervention targets.

## Figures and Tables

**Table 1:** Diarrhoea associated with FDA approved TKIs targeting EGFR.

Drug	Target	All grade diarrhoea	Grade 3/4 diarrhoea	Reference
Erlotinib (Tarceva)	EGFR	62–76%	3–10%	[8]
Gefitinib (Iressa)	EGFR	57–67%	12–13%	[8]
Lapatinib (Tykerb)	EGFR, HER2	50%	6%	[12]
Vandetanib (Caprelsa)	EGFR, VEGFR	56–67%	10%	[13, 14]
Afatinib (Gilotrif)	EGFR, HER2, and ErbB4	78%	14%	[8]

## References

[1] Alimonti A, Gelibter A, Pavese I (2004). New approaches to prevent intestinal toxicity of irinotecan-based regimens. *Cancer Treatment Reviews*.

[2] Gibson RJ, Keefe DMK (2006). Cancer chemotherapy-induced diarrhoea and constipation: mechanisms of damage and prevention strategies. *Supportive Care in Cancer*.

[3] Sharma R, Tobin P, Clarke SJ (2005). Management of chemotherapy-induced nausea, vomiting, oral mucositis, and diarrhoea. *The Lancet Oncology*.

[4] Kitagawa D, Yokota K, Gouda M (2013). Activity-based kinase profiling of approved tyrosine kinase inhibitors. *Genes to Cells*.

[5] Keefe DMK, Gibson RJ (2007). Mucosal injury from targeted anti-cancer therapy. *Supportive Care in Cancer*.

[6] Keefe D, Anthony L (2008). Tyrosine kinase inhibitors and gut toxicity: a new era in supportive care. *Current Opinion in Supportive and Palliative Care*.

[7] Thomas SK, Fossella FV, Liu D (2006). Asian ethnicity as a predictor of response in patients with non-small-cell lung cancer treated with gefitinib on an expanded access program. *Clinical Lung Cancer*.

[8] Hirsh V (2011). Managing treatment-related adverse events associated with EGFR tyrosine kinase inhibitors in advanced non-small-cell lung cancer. *Current Oncology*.

[9] Chen P, Wang L, Liu B, Zhang H, Liu H, Zou Z (2011). EGFR-targeted therapies combined with chemotherapy for treating advanced non-small-cell lung cancer: a meta-analysis. *European Journal of Clinical Pharmacology*.

[10] Cohen EEW, Halpern AB, Kasza K, Kocherginsky M, Williams R, Vokes EE (2009). Factors associated with clinical benefit from epidermal growth factor receptor inhibitors in recurrent and metastatic squamous cell carcinoma of the head and neck. *Oral Oncology*.

[11] Petrelli F, Borgonovo K, Cabiddu M, Lonati V, Barni S (2012). Relationship between skin rash and outcome in non-small-cell lung cancer patients treated with anti-EGFR tyrosine kinase inhibitors: a literature-based meta-analysis of 24 trials. *Lung Cancer*.

[12] Crown JP, Burris HA, Boyle F (2008). Pooled analysis of diarrhea events in patients with cancer treated with lapatinib. *Breast Cancer Research and Treatment*.

[13] Chau NG, Haddad RI (2013). Vandetanib for the treatment of medullary thyroid cancer. *Clinical Cancer Research*.

[14] Morabito A, Piccirillo MC, Falasconi F (2009). Vandetanib (ZD6474), a dual inhibitor of vascular endothelial growth factor receptor (VEGFR) and epidermal growth factor receptor (EGFR) tyrosine kinases: current status and future directions. *The Oncologist*.

[15] Loriot Y, Perlemuter G, Malka D, Penault-Llorca F, Boige V, Deutsch E (2008). Drug insight: gastrointestinal and hepatic adverse effects of molecular-targeted agents in cancer therapy. *Nature Clinical Practice. Oncology*.

[16] Burris HA, Hurwitz HI, Dees EC (2005). Phase I safety, pharmacokinetics, and clinical activity study of lapatinib (GW572016), a reversible dual inhibitor of epidermal growth factor receptor tyrosine kinases, in heavily pretreated patients with metastatic carcinomas. *Journal of Clinical Oncology*.

[17] Abbas R, Hug BA, Leister C, Sonnichsen D (2012). A double-blind, randomized, multiple-dose, parallel-group study to characterize the occurrence of diarrhea following two different dosing regimens of neratinib, an irreversible pan-ErbB receptor tyrosine kinase inhibitor. *Cancer Chemotherapy and Pharmacology*.

[18] Rudin CM, Liu W, Desai A (2008). Pharmacogenomic and pharmacokinetic determinants of erlotinib toxicity. *Journal of Clinical Oncology*.

[19] Berlanga-Acosta J, Playford RJ, Mandir N, Goodlad RA (2001). Gastrointestinal cell proliferation and crypt fission are separate but complementary means of increasing tissue mass following infusion of epidermal growth factor in rats. *Gut*.

[20] Harandi A, Zaidi AS, Stocker AM, Laber DA (2009). Clinical efficacy and toxicity of anti-EGFR therapy in common cancers. *Journal of Oncology*.

[21] McCole DF, Barrett KE (2009). Decoding epithelial signals: Critical role for the epidermal growth factor receptor in controlling intestinal transport function. *Acta Physiologica*.

[22] Medina PJ, Goodin S (2008). Lapatinib: a dual inhibitor of human epidermal growth factor receptor tyrosine kinases. *Clinical Therapeutics*.

[23] Keisner SV, Shah SR (2011). Pazopanib: the newest tyrosine kinase inhibitor for the treatment of advanced or metastatic renal cell carcinoma. *Drugs*.

[24] van Erp NP, Gelderblom H, Guchelaar HJ (2009). Clinical pharmacokinetics of tyrosine kinase inhibitors. *Cancer Treatment Reviews*.

[25] Boerma M, Wang J, Burnett AF, Santin AD, Roman JJ, Hauer-Jensen M (2007). Local administration of interleukin-11 ameliorates intestinal radiation injury in rats. *Cancer Research*.

[26] Bowen JM, Stringer AM, Gibson RJ, Yeoh AS, Hannam S, Keefe DM (2007). VSL#3 probiotic treatment reduces chemotherapy-induced diarrhea and weight loss. *Cancer Biology & Therapy*.

[27] Watkins B, Pouliot K, Fey E, Tuthill C, Sonis S (2010). Attenuation of radiation-and chemoradiation-induced mucositis using gamma-d-glutamyl-l-tryptophan (SCV-07). *Oral Diseases*.

[28] Bowen JM, Mayo BJ, Plews E (2012). Development of a rat model of oral small molecule receptor tyrosine kinase inhibitor-induced diarrhea. *Cancer Biology and Therapy*.

[29] Rusnak DW, Lackey K, Affleck K (2001). The effects of the novel, reversible epidermal growth factor receptor/ErbB-2 tyrosine kinase inhibitor, GW2016, on the growth of human normal and tumor-derived cell lines in vitro and in vivo. *Molecular Cancer Therapeutics*.

[30] Henson ES, Gibson SB (2006). Surviving cell death through epidermal growth factor (EGF) signal transduction pathways: Implications for cancer therapy. *Cellular Signalling*.

[31] Ryan Q, Ibrahim A, Cohen MH (2008). FDA drug approval summary: lapatinib in combination with capecitabine for previously treated metastatic breast cancer that overexpresses HER-2. *The Oncologist*.

[32] Burris HA (2004). Dual kinase inhibition in the treatment of breast cancer: initial experience with the EGFR/ErbB-2 inhibitor lapatinib. *Oncologist*.

[33] Baselga J, Bradbury I, Eidtmann H (2012). Lapatinib with trastuzumab for HER2-positive early breast cancer (NeoALTTO): a randomised, open-label, multicentre, phase 3 trial. *The Lancet*.

[34] Azim HA, Agbor-Tarh D, Bradbury I (2013). Pattern of rash, diarrhea, and hepatic toxicities secondary to lapatinib and their association with age and response to neoadjuvant therapy: analysis from the NeoALTTO trial. *Journal of Clinical Oncology*.

[35] Burris HA, Taylor CW, Jones SF (2009). A phase I and pharmacokinetic study of oral lapatinib administered once or twice daily in patients with solid malignancies. *Clinical Cancer Research*.

[36] Koch KM, Reddy NJ, Cohen RB (2009). Effects of food on the relative bioavailability of lapatinib in cancer patients. *Journal of Clinical Oncology*.

[37] Hare KJ, Hartmann B, Kissow H, Holst JJ, Poulsen SS (2007). The intestinotrophic peptide, GLP-2, counteracts intestinal atrophy in mice induced by the epidermal growth factor receptor inhibitor, gefitinib. *Clinical Cancer Research*.

[38] Yusta B, Holland D, Koehler JA (2009). ErbB signaling is required for the proliferative actions of GLP-2 in the murine gut. *Gastroenterology*.

[39] Rasmussen AR, Viby N, Hare KJ (2010). The intestinotrophic peptide, GLP-2, counteracts the gastrointestinal atrophy in mice induced by the epidermal growth factor receptor inhibitor, erlotinib, and cisplatin. *Digestive Diseases and Sciences*.

[40] Mitchell EP (2006). Gastrointestinal toxicity of chemotherapeutic agents. *Seminars in Oncology*.

[41] Li J, Karlsson MO, Brahmer J (2006). CYP3A phenotyping approach to predict systemic exposure to EGFR tyrosine kinase inhibitors. *Journal of the National Cancer Institute*.

[42] Lu J-F, Eppler SM, Wolf J (2006). Clinical pharmacokinetics of erlotinib in patients with solid tumors and exposure-safety relationship in patients with non-small cell lung cancer. *Clinical Pharmacology and Therapeutics*.

[43] Gibson RJ, Bowen JM, Inglis MRB, Cummins AG, Keefe DMK (2003). Irinotecan causes severe small intestinal damage, as well as colonic damage, in the rat with implanted breast cancer. *Journal of Gastroenterology and Hepatology*.

[44] Gibson RJ, Bowen JM, Keefe DMK (2005). Palifermin reduces diarrhea and increases survival following irinotecan treatment in tumor-bearing DA rats. *International Journal of Cancer*.

[45] Logan RM, Stringer AM, Bowen JM, Gibson RJ, Sonis ST, Keefe DMK (2009). Is the pathobiology of chemotherapy-induced alimentary tract mucositis influenced by the type of mucotoxic drug administered?. *Cancer Chemotherapy and Pharmacology*.

[46] Bateman E, Bowen J, Stringer A (2013). Investigation of effect of nutritional drink on chemotherapy-induced mucosal injury and tumor growth in an established animal model. *Nutrients*.

[47] Cohen RB (2014). Current challenges and clinical investigations of epidermal growth factor receptor (EGFR)—and ErbB family-targeted agents in the treatment of head and neck squamous cell carcinoma (HNSCC). *Cancer Treatment Reviews*.

